# Enabling Ultrafine Ru Nanoparticles with Tunable Electronic Structures via a Double-Shell Hollow Interlayer Confinement Strategy toward Enhanced Hydrogen Evolution Reaction Performance

**DOI:** 10.1021/acs.nanolett.3c03514

**Published:** 2023-12-01

**Authors:** Xiaoyan Liu, Lihua Gong, Liwei Wang, Chaoqun Chang, Panpan Su, Yuhai Dou, Shi Xue Dou, Ying Li, Feilong Gong, Jian Liu

**Affiliations:** † Key Laboratory of Surface and Interface Science and Technology of Henan Province, College of Material and Chemical Engineering, Zhengzhou University of Light Industry, Zhengzhou, Henan 450001, PR China; ‡ State Key Laboratory of Catalysis, Dalian Institute of Chemical Physics, Chinese Academy of Sciences, Dalian, Liaoning 116023, PR China; § Institute of Industrial Catalysis, Zhejiang University of Technology, Hangzhou Chaowang Road 18, Hangzhou, Zhejiang 310014, PR China; ∥ DICP-Surrey Joint Centre for Future Materials, Department of Chemical and Process Engineering and Advanced Technology Institute of University of Surrey, Guildford, Surrey GU2 7XH, U.K.; ⊥ College of Chemistry and Chemical Engineering, Inner Mongolia University, Hohhot, Inner Mongolia 010021, PR China; # Institute of Energy Materials Science, University of Shanghai for Science and Technology, Shanghai 200093, PR China

**Keywords:** electronic structure, nanoreactor, confinement effect, hollow carbon sphere, water splitting

## Abstract

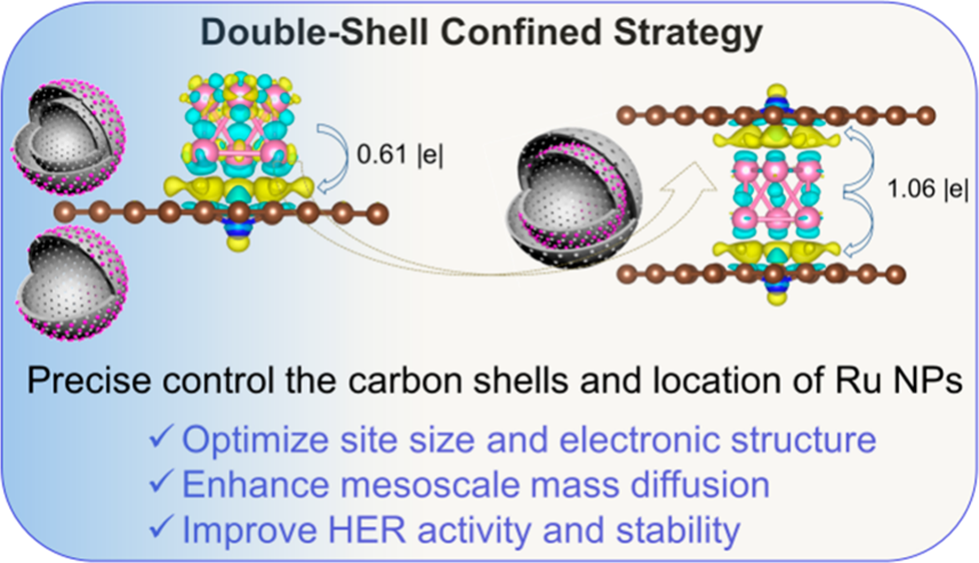

Engineering of the catalysts’ structural stability and electronic structure could enable high-throughput H_2_ production over electrocatalytic water splitting. Herein, a double-shell interlayer confinement strategy is proposed to modulate the spatial position of Ru nanoparticles in hollow carbon nanoreactors for achieving tunable sizes and electronic structures toward enhanced H_2_ evolution. Specifically, the Ru can be anchored in either the inner layer (Ru-DSC-I) or the external shell (Ru-DSC-E) of double-shell nanoreactors, and the size of Ru is reduced from 2.2 to 0.9 nm because of the double-shell confinement effect. The electronic structures are efficiently optimized thereby stabilizing active sites and lowering the reaction barrier. According to finite element analysis results, the mesoscale mass diffusion can be promoted in the double-shell configuration. The Ru-DSC-I nanoreactor exhibits a much lower overpotential (η_10_ = 73.5 mV) and much higher stability (100 mA cm^–2^). Our work might shed light on the precise design of multishell catalysts with efficient refining electrostructures toward electrosynthesis applications.

Electrochemical hydrogen evolution reaction (HER) is one promising zero-carbon footprint method for the production of green hydrogen and is also an important half reaction of energy devices.^1−3^ Many strategies have been proposed to lower the overpotentials of the catalysts for achieving high-activity HER in acidic and alkaline environments.^4−9^ Pt-based materials are the best electrocatalysts for alkaline HER so far, whereas they are very scarce and expensive, which limits their broad commercial applications.^10^ Numerous research efforts have been devoted to reducing the Pt consumption or replacing Pt with other materials.^11−14^ It is worth noting that Ru is regarded as an ideal candidate due to its relatively low cost but similar hydrogen binding energy to Pt.^15,16^ Combining ultrafine Ru with carbon is demonstrated to be a promising way to tailor catalytic activity due to the strong catalyst–support interaction.^16,17^ However, the higher cohesive energy of Ru in comparison with Pt makes Ru prefer to aggregate, and strong Ru–H bonds render dissociation difficult, resulting in an unsatisfactory HER process to date.

Generally, the apparent rate of a surface reaction occurring on a heterogeneous catalyst is strongly dependent on its geometric properties and electronic structures, which act together in determining adsorption of intermediates, activation energies, and energy barriers for the reaction.^8,18−20^ This complexity hugely promotes the elaborated design of novel Ru-based catalysts via finding a suitable support to increase the number of active sites and improve the intrinsic activity of each single site.^4,21−25^ Controlled construction of catalysts with well-designed structures and nanoarchitectures could potentially increase and activate catalytic sites due to the large exposed surface atoms and strong metal–support interaction.^26−32^ Core–shell and hollow structures are powerful platforms for controlled release, confined nanocatalysis, and optical and electronic applications.^28,33−37^ It has been reported that hollow structured carbon could accelerate, decelerate, or inhibit conversion of the intermediate in the void space.^38−41^ Therefore, the unique hollow carbon is expected to facilitate the exposure of active sites to reagents and enhance the accessibility of reactant.^42,43^

Nanoreactors, particularly combining metal nanoparticles and hollow nanomaterials, have many advantages in comparison with conventional catalysts for loading metal nanoparticles on bulk support: (i) Each nanoparticle isolated by a shell has a relatively homogeneous environment around the particle surface. The outer shell structure also hinders the aggregation of neighboring particles, even under harsh reaction conditions; (ii) the interaction between metal and support is more effective than that of the bulk forms, feasibly leading to highly catalytic activity. Nanoreactors have great potential applications in various catalytic reactions.^12−14,44−47^ For instance, a nanoreactor framework of a Au@SiO_2_ yolk–shell structure has been applied for the catalytic reduction of *p*-nitrophenol.^48^ Another nanoreactor composed of Ni@N-CNCs has shown superior activity for ORR.^49^ By tuning the morphology, size, and composition of the catalyst, the key factors governing the catalytic activity can be optimized, resulting in excellent HER catalysts.^50^ Through precise control of the microenvironment, reaction channels, and active components of the nanoreactor, the activity, selectivity, and reaction pathway could be adjusted accurately. Hollow double-shell nanospheres have received increasing attention as they can be used as the most ideal framework in a nanoreactor for electrocatalysis.^51^ Though strategies such as dopants, vacancies, heterostructures, etc. have been used to regulate the electronic structures of the above catalysts, it still remains a challenge to achieve hollow double-shell nanoreactors with active sites at a certain spatial position, which might be beneficial to the catalytic activity and durability.

Herein, we report a double-shell interlayer confinement strategy to precisely modulate the spatial position of Ru nanoparticles (NPs) in hollow carbon spheres. The Ru NPs are anchored in the inner or external shell of double-shell carbon spheres to produce the Ru-DSC-I and Ru-DSC-E nanoreactors. The particle size and electronic structures are efficiently tailored in Ru-DSC-I, which contributes to the improved mass activity and stability. The finite element analysis (FEA) results demonstrate that the double-shell structure is more conducive to the mesoscale diffusion of electrolyte. Benefiting from the ultrafine size, optimized electronic structures, and enhanced mesoscale diffusion, Ru-DSC-I presents the superior HER activity and stability.

The spatial positions of Ru nanoparticles in the hollow carbon spheres are precisely modulated via a double-shell-confined strategy to produce a group of unique nanoreactors (Scheme S1 and Figure 1a). Specifically, the cavity is formed by using a polystyrene sphere (PS) core, and 3-aminophenol-formaldehyde resin (APF) is utilized as a nitrogen-doped carbon shell precursor. To obtain the double-shell structure, a silica middle shell is introduced between the two APF shells, and the two APF-derived nitrogen-doped carbon shells are completely separated after the carbonization and silica etching process. To precisely locate the Ru on the interior or external shell, Ru^3+^ is adsorbed by the inner and external APF shell, respectively, while the intense coordination between Ru^3+^ and the NH_2_ group ensures the location of the Ru species. Thus, the Ru-DSC-I and Ru-DSC-E are successfully prepared via altering the order of adsorption of Ru^3+^ in the multistep APF and silica coating process. The hollow single-shell nanoreactor (Ru-SSC) is also synthesized by using a similar method for comparison, but without adding the second APF shell coating.

**Figure 1:**
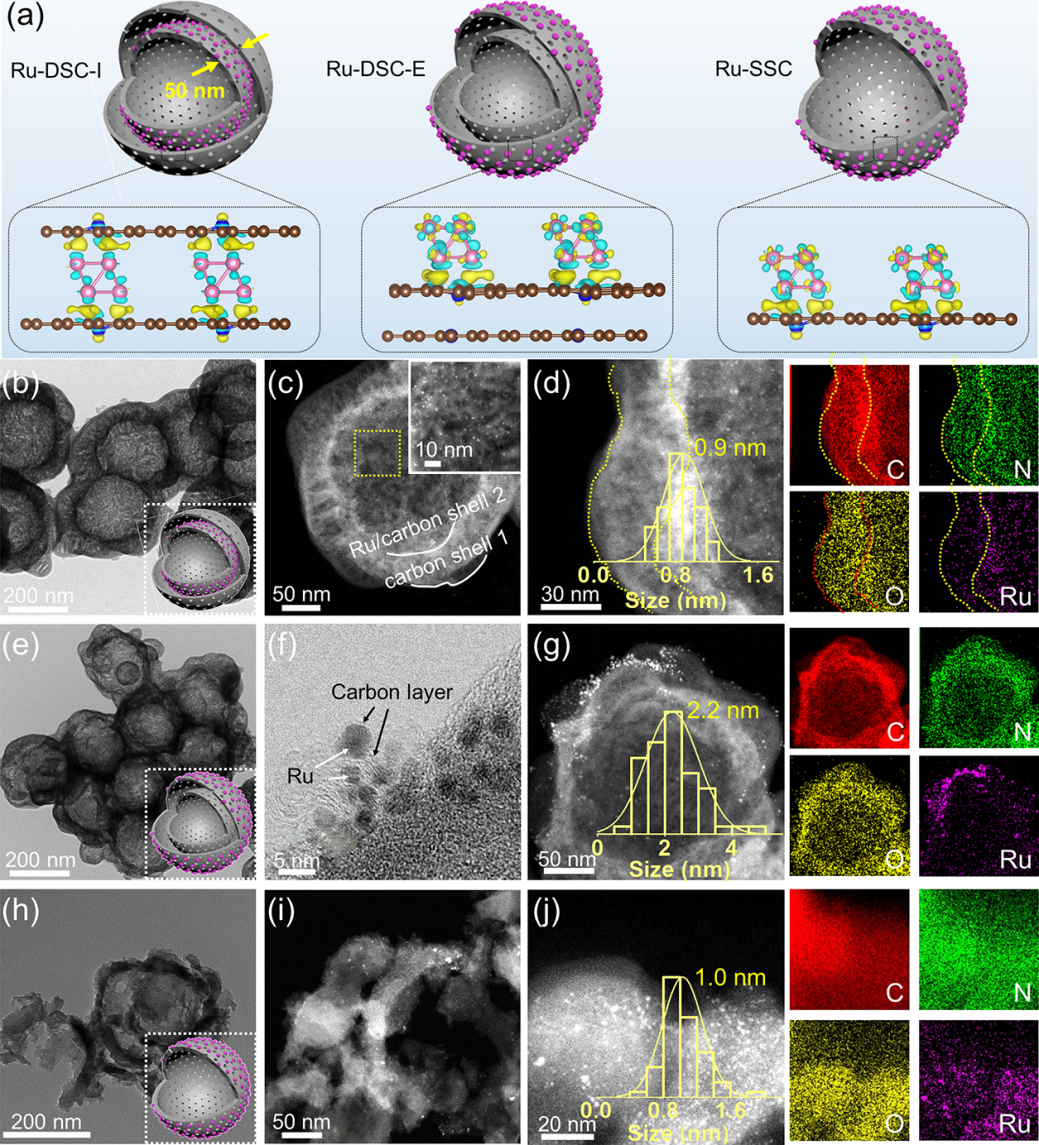
Schematic illustration and morphology characterizations. (a) Schematic illustration of the Ru-SSC, Ru-DSC-I, and Ru-DSC-E. (b) TEM image, (c) HAADF-HRTEM image, and (d) enlarged HAADF-HRTEM image of the selected area and corresponding elemental mapping images of Ru-DSC-I. (e) TEM image, (f) HRTEM image, and (g) HAADF-HRTEM image and corresponding elemental mapping images of Ru-DSC-E. (h) TEM image, (i) HAADF-HRTEM image, and (j) enlarged HAADF-HRTEM image of the selected area and corresponding elemental mapping images of Ru-SSC. Insets of (d), (g), and (j) are size distributions of Ru NPs. The scale bar in inset of (c) is 10 nm.

Transmission electron microscopy (TEM) images of the PS core showed monodispersed nanospheres with a uniform size of 200 nm (Figure S1a). The TEM image of the PS@APF (Figure S1b) spheres revealed that the APF shell with a thickness of ca. 25 nm is coated onto the PS core to form a raspberry-like morphology. After the adsorption of RuCl_3_, there is no morphology difference between PS@APF and PS@APF-Ru (Figure S1c). From Figure S1d,e, after SiO_2_ and the second APF shell coating, the raspberry-like morphology changed to relatively smooth. During the pyrolysis step, the PS core decomposed completely to form the hollow void structure (Figure S2). Followed by the SiO_2_ shell removal, the double-shell N-doped carbon with a width of 50 nm was obtained between the two carbon shells (Figure 1b). From the TEM and high-angle annular dark-field (HAADF) images (Figure 1), the location of Ru NPs can be readily controlled. For the Ru^3+^ adsorbed to the first APF-coated shell, the uniform Ru NPs are embedded only in the inner carbon shell (Figure 1c,d), indicating that the Ru NPs are apparently only located on the inner carbon shell; more detailed images can be founded in Figure S3. Additionally, the ultrafine Ru NPs were well dispersed, with a mean size of 0.9 nm (inset of Figure 1d). Furthermore, the energy-dispersive X-ray spectroscopy (EDS) mapping images of the Ru-DSC-I also confirmed that the Ru NPs were dispersed in the inner carbon shell (the purple images). The preparation of Ru-DSC-E only altered the Ru^3+^ adsorption to the second APF-coated shell, and the detailed diagram can be seen in Scheme S1. The dual-shell structure is revealed (Figure 1e), and the Ru NPs are only distributed in the external shell surface (Figure 1g and Figure S4). However, the mean size of Ru increased to 2.2 nm (inset of Figure 1g). Moreover, the HRTEM image (Figure 1f) reveals that the Ru NPs are covered with a thin carbon layer, demonstrating that the silica shell could guarantee the exposure of Ru NPs.

For the single-shell nanoreactor (Ru-SSC), the TEM and HAADF-HRTEM images (Figure 1h–j) verify the half-bowl-like carbon shell due to the fast decomposition of the PS core, which destroyed the thin single carbon shell after carbonization and the SiO_2_ etching step. And the mean size of the Ru NPs was calculated to be 1.1 nm (inset of Figure 1j). The smaller Ru NP size for Ru-DSC-I and Ru-SSC could be attributed to the confinement effect of the silica shell during the carbonization process and the nitrogen atoms with lone pairs of electrons serving as the sites for Ru nucleation, thereby stabilizing the ultrafine Ru NPs. For Ru-DSC-E, only nitrogen atoms were utilized to stabilize the Ru NPs leading to larger Ru NPs. From the EDS results, the elements including C, N, O, and Ru distributed uniformly across the entire catalyst, while Ru existed at the inner shell or external shell, respectively. This finding is consistent with our tailored strategy that Ru NPs can be precisely confined in different locations.

To explore the pyrolysis process of PS@APF, PS@APF@SiO_2_, and PS@APF@SiO_2_@APF, the thermal gravimetric analysis (TGA) results were collected (Figure 2a). For all samples, there are two weight loss peaks at 227 °C and 408–432 °C. The first peak at 227 °C can be assigned to the decomposition of the PS core, and the weight loss was 25 wt % which was equal to the mass of polystyrene. And when the temperature increases subsequently to 432 °C, a conspicuous weight loss of 50 wt % is observed for PS@APF composites, probably due to the pyrolysis of the APF resin.^52^ An obvious shift in pyrolysis temperature for the APF resin between three samples is the pyrolysis temperature from 432 to 415 °C and to 408 °C after another APF resin coating, which means that the confined structure could lower the pyrolysis temperature.

**Figure 2:**
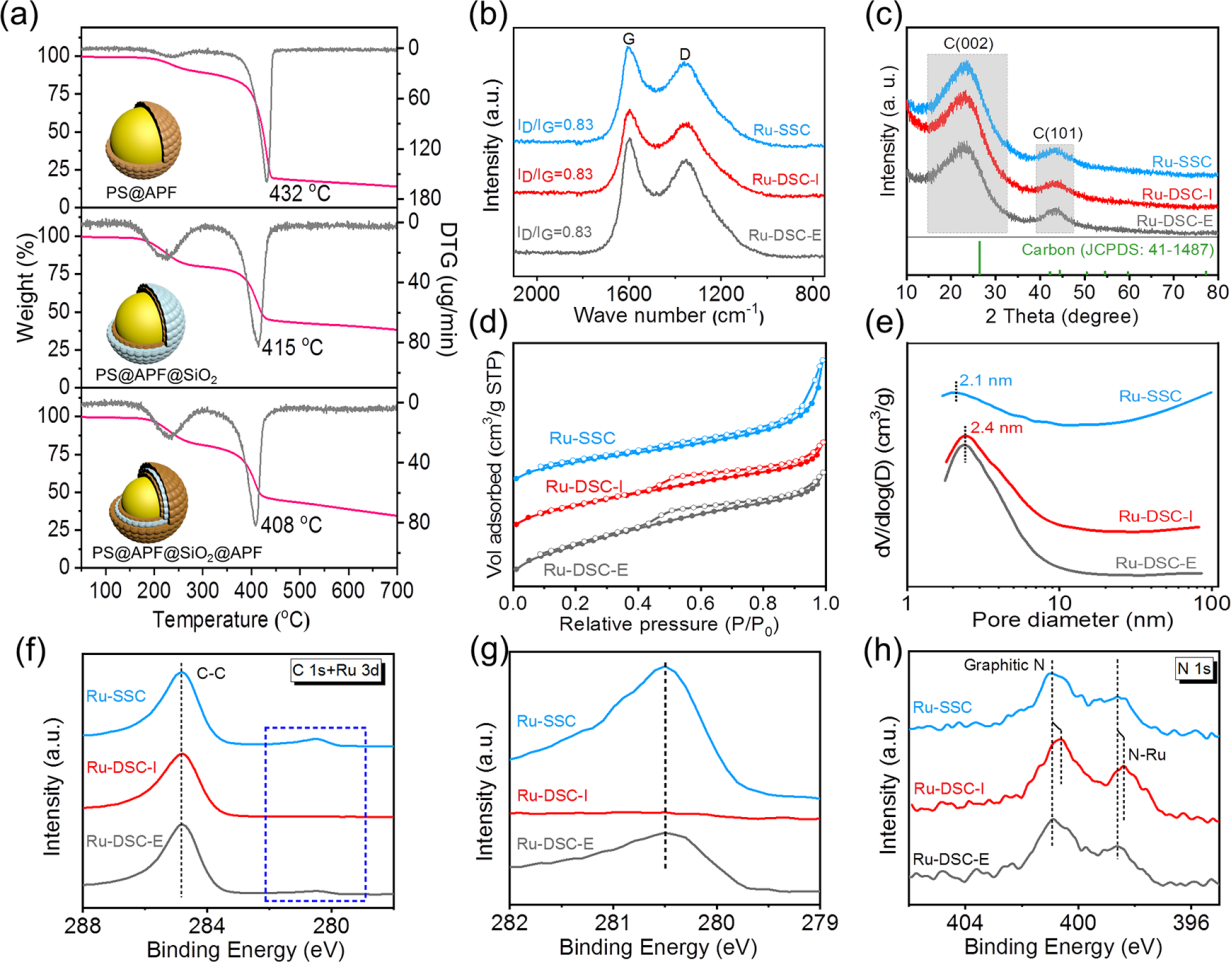
Structure characterizations of the catalysts. (a) TG and DTG curves of PS@APF (top), PS@APF@SiO_2_ (medium), and PS@APF@SiO_2_@APF (bottom); conditions: N_2_, 30 mL min^–1^, 5 °C min^–1^. (b) Raman spectra, (c) XRD patterns, (d) N_2_ adsorption–desorption isotherm, (e) pore diameter distribution, (f, g) high-resolution XPS spectra of C 1s and Ru 3p, and (h) high-resolution XPS spectra of N 1s for the Ru-SSC, Ru-DSC-I, and Ru-DSC-E.

From the Raman spectrum (Figure 2b), two peaks named the D and G bands correspond to the nongraphitic and graphitic carbon, respectively. The same I_D_/I_G_ ratio (0.83) suggested that they had a similar graphitization degree. According to the X-ray diffraction (XRD) results (Figure 2c and Figure S6), two broad peaks appeared for all samples, which corresponded to (002) and (101) facets from the graphitic carbon. Besides, no collective characteristic peaks for aggregation of Ru NPs confirmed the high dispersion. In addition, the Ru content of all prepared catalysts was measured to be around 1.5 wt % by inductively coupled plasma–atomic emission spectrometry (ICP-AES) (Table S1).

From the N_2_ adsorption–desorption isotherm, the pure double-shell carbon spheres, Ru-DSC-I, and Ru-DSC-E, delivered a shape of the IV-type isotherm with a H4 hysteresis loop, demonstrating that these samples could possess cavities (Figure 2d and Figure S7). By contrast, there was no hysteresis loop for Ru-SSC, indicating a lack of the cavity. Accordingly, the BET specific areas were calculated to be 676 m^2^ g^–1^ for the Ru-DSC-I and 631 m^2^ g^–1^ for the Ru-DSC-E, which were much larger than 494 m^2^ g^–1^ of the Ru-SSC (Table S1). The small mesopore size (<3 nm) was produced owing to the template of CTAB and thus cross-linking properties of APF precursors, as well as a large portion of the resin polymer frameworks during carbonization process (Figure 2e).

From the X-ray photoelectron spectroscopy (XPS) spectra of Ru 3d, the peak at 280.5 eV verified the metallic state of Ru in the Ru-SSC and Ru-DSC-E, while no peak belonging to Ru can be found in the Ru-DSC-I (Figure 2f,g, Figure S8). The undetectable Ru for the Ru-DSC-I could be attributed to the Ru nanoparticles being in the inner shell of double-shell carbon where the X-ray cannot detect them (50 nm, Figure 1c,d). In addition, the peak locations of graphitic N and N-Ru were unchanged for the Ru-SSC and Ru-DSC-E (Figure 2h), while they presented negative shifts of 0.3 and 0.2 eV in the Ru-DSC-I, respectively, meaning that anchoring Ru in the inner shell of the double-shell support could generate much stronger interfacial interaction. Figure S9 disclosed that the contact angles of Ru-SSC and Ru-DSC-I were 20° and 11°, respectively. The excellent hydrophilicity would facilitate the closed contact between the liquid electrolyte and active sites.

Seen from the polarization curves collected in alkaline electrolyte (1 M KOH) (Figure 3a), η_10_, an important parameter of the HER activity, can be identified at −73.5 mV for Ru-DSC-I. Ru-DSC-I showed the lowest overpotential value, even better than the commercial Pt/C. Additionally, the activity of Ru-DSC-I was also much higher than those of noble metal based catalysts reported in the literature (Figure 3b, Table S2). Ru-DSC-I delivered much smaller Tafel slopes of 83.7 mV dec^–1^ compared with those of Pt/C, Ru-DSC-E, and Ru-SSC (Figure S10). According to the chronoamperometry method, all of the nanoreactors remained stable at 10 mA cm^–2^ (Figure 3c). Beyond that, at a high current density of 100 mA cm^–2^, the Ru-DSC-I exhibited almost unchanged plots for 24 h, and the stability of Ru-SSC and Ru-DSC-E gradually decreased with decay rates of 1.68 and 0.91 mA cm^–2^ h^–1^, respectively. ICP-AES was employed to check the Ru content after catalysis (Table S3). The weight percentage of Ru in the Ru-DSC-I was ca. 1.46 wt %, which was maintained almost constant in comparison with the fresh catalyst. Nevertheless, Ru in the Ru-SSC and Ru-DSC-E preferred to escape from the support. Thus, the superior stability of the Ru-DSC-I nanoreactor at high voltage could be attributed to the particular spatial position of Ru.

**Figure 3:**
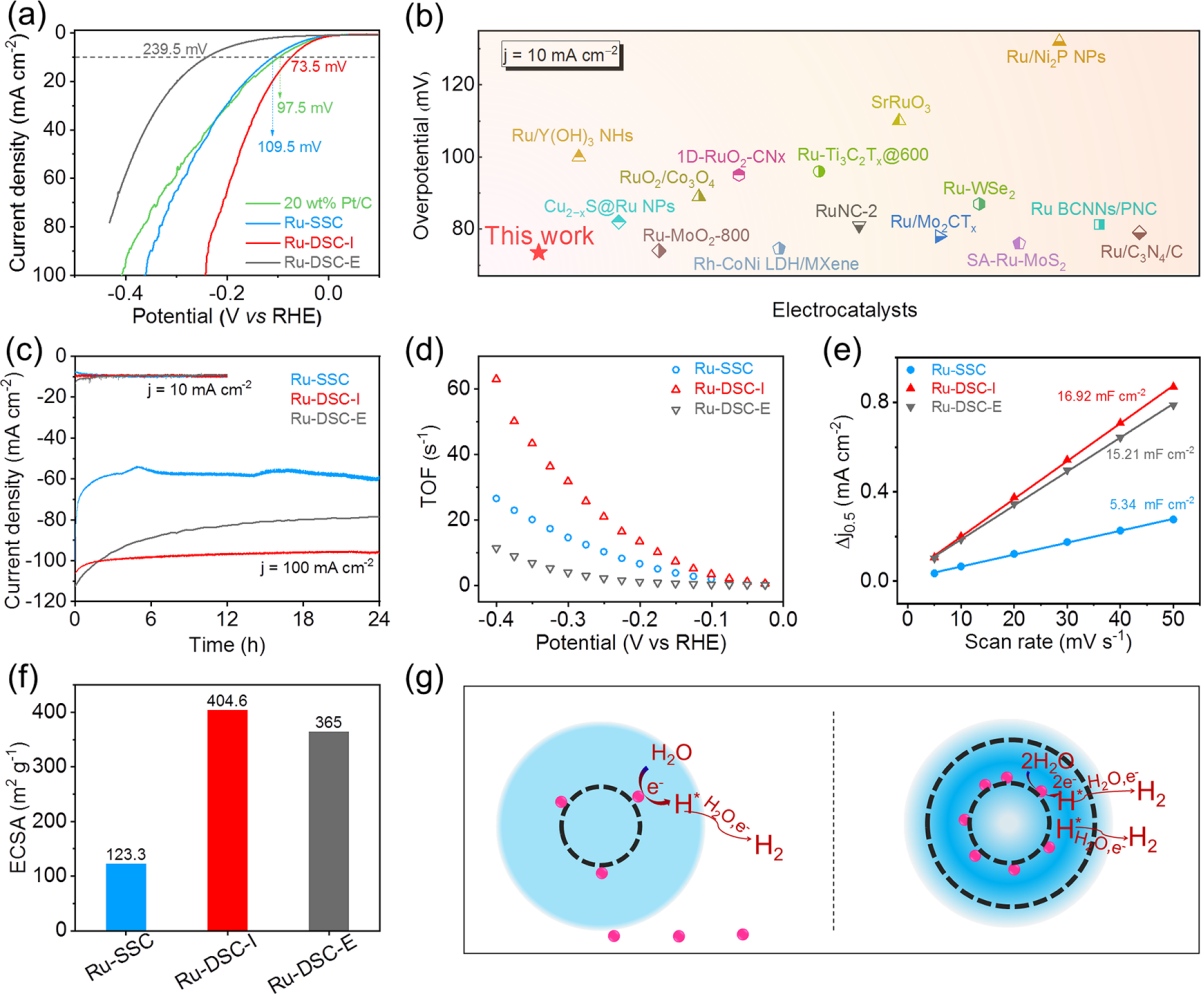
Electrochemical measurements of Ru-SSC, Ru-DSC-I, Ru-DSC-E, and commercial Pt/C. (a) The comparison of polarization curves. (b) The comparison of overpotential for the Ru-DSC-I and noble metal based catalysts. (c) The comparison for stability at 10 and 100 mA cm^–2^. (d) The comparison for TOF values. (e) Differences ion current density (Δj = ja – jc) plotted against rates at 0.55 V. (f) ECSA values. (g) Potential forming mechanism of the double-shell promoting electron transfer effect. Catalyst loading: 0.17 mg cm^–2^ for Ru-SSC, Ru-DSC-I, and Ru-DSC-E; 0.0127 mg cm^–2^ for commercial 20 wt % Pt/C.

Seen from the turnover frequency (TOF) shown in Figure 3d, Ru-DSC-I exhibited a value of 63 s^–1^ at 400 mV, which is 2.3-fold and 5.7-fold higher than Ru-SSC and Ru-DSC-E, verifying its exceptional activity. To further investigate the intrinsic specific activity, the double layer capacitances (C_dl_) were evaluated from the measured CV curves (Figure S11). The C_dl_ values were calculated to be 16.92, 15.21, and 5.34 mF cm^–2^ for Ru-DSC-I, Ru-DSC-E, and Ru-SSC, respectively, implying more accessible active sites for the Ru-DSC-I (Figure 3e). In addition, there were remarkable differences in the electrochemical active surface area (ECSA) with a diminishing order of Ru-DSC-I > Ru-DSC-E > Ru-SSC (Figure 3f). The highest ECSA of Ru-DSC-I was attributed to the double-shell structure, as Ru-DSC-E with the same double shell structure exhibited a comparable ECSA. These results indicate the double-layer structure endows the Ru-DSC-I catalyst with high intrinsic activity. Nyquist plots were collected from electrochemical impedance spectroscopy (EIS) measurements (Figure S12). The Ru-DSC-I presented the smallest semicircle residence of 8.10 Ω in comparison with Ru-DSC-E (8.86 Ω) and Ru-SSC (23.9 Ω), corresponding to the lowest charge transfer resistance (*R*_ct_), indicating a faster ion transfer in the Ru-DSC-I catalyst. Based on the above results, the possible potential mechanism is described in Figure 3g. As demonstrated by the size dimension, the small diameter of Ru NPs in the double-shelled structure endows catalysts with more active sites, thus effectively accelerating the HER process. Simultaneously, the electron state of the Ru NPs in the confinement environment of the double-shelled structure was very likely modified, which may promote the hydrogen desorption. Additionally, the mass transfer of the electrolyte in this microenvironment may be faster, further enhancing catalytic activity of Ru-DSC-I. In addition, the confinement effect of the double-shell avoided the loss of Ru active sites, guaranteeing the durability of the catalysts.

To theoretically elucidate the above mechanism, DFT calculations were performed to investigate the effect of the spatial position. The Ru@C-S model was representative of the Ru-SSC and Ru-DSC-E, and the Ru@C-I model corresponded to the Ru-DSC-I (Figure S15). From the total density of states (TDOS) and projected density of states (PDOS) (Figures S13–14), the Ru 3d state dominated the prominent state, and the intensity of Ru@C-I was much stronger near the Fermi level, implying higher conductivity. The d-band center of Ru was calculated to be −3.70 eV for Ru@C-I (Figure 4a), which was much lower than −2.54 eV of Ru@C-S. By contrast, the downshifted d-band center meant more electron filling of the antibonding states in the Ru@C-I, endowing it with easier hydrogen desorption than Ru@C-S. In addition, the electron density differences (EDDs) (Figure 4b) revealed that the Ru@C-I could create a double interface, which caused more charge aggregation at the interface. The Bader analysis revealed that the transfer electron of Ru@C-I was 1.06 |e| from Ru to the carbon substrate, which was ca. 1.6 times higher than that of Ru@C-S (0.61 |e|). The EDD results suggested that more electron redistribution occurred in the double-shell structure, which was beneficial for activating the interface. The adsorption energy of Ru in Ru@C-I was calculated to be −3.2 eV, showing an ca. 2-fold improvement compared with that of Ru@C-S (Figure 4c). The enhanced adsorption energy suggested that the stability of Ru could be improved when it was anchored in the inner of double-layered carbon, which was consistent with the stability measurement (Figure 4c).

**Figure 4:**
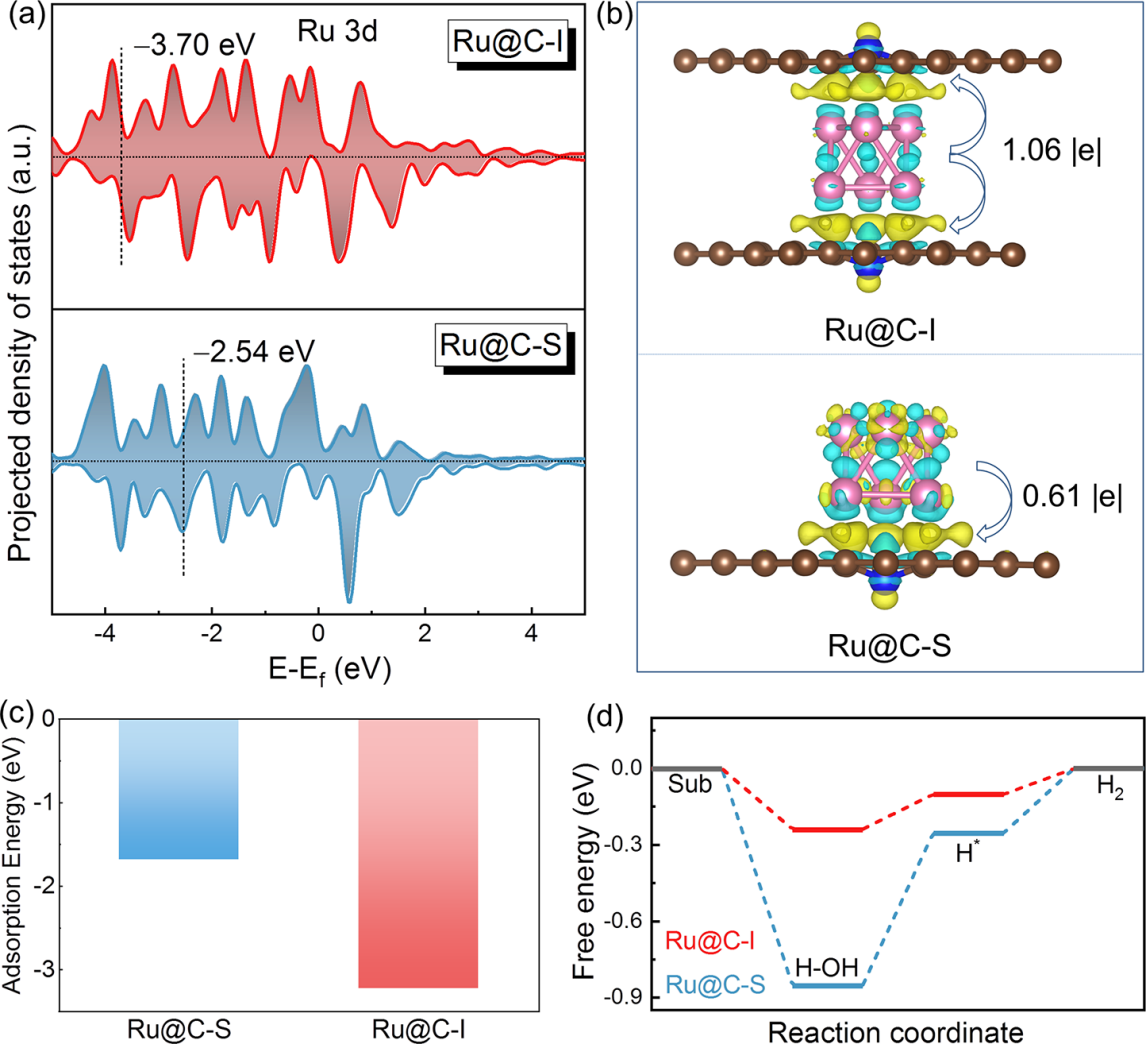
DFT calculations for the Ru@C-I and Ru@C-S. (a) PDOS of Ru 3d. (b) EDDs and Bader analysis. (c) The adsorption energies of Ru in the different supports. (d) Free energy diagrams. Yellow and cyan colors in (b) are charge aggregation and depletion in the 0.005 e/Bohr^3^ isosurface, respectively.

The energy barriers of hydrogen evolution were also calculated to study the HER process in Ru@C-I and Ru@C-S. The corresponding reaction pathways and optimized structures in the different models are shown in Figure 4d and Figure S15. The energy barriers for the water dissociation step and H_2_ generation step of the Ru-DSC-I were calculated to be −0.24 and −0.10 eV, respectively, both of which were much closer to 0 eV, in comparison with the values of −0.85 and −0.25 eV of Ru@C-S. The calculated results further verified that the as-prepared Ru-DSC-I nanoreactor remarkably facilitated water dissociation and H* desorption. Based on the above calculations, the electronic structures of the catalyst and the reaction barriers of hydrogen evolution could be efficiently moderated via regulating the spatial position of Ru in the confined carbon support, thereby tailoring the HER performances.

The FEA was further used to simulate the mesoscale mass transfer properties in the single-shell and double-shell carbon models. Figure 5a,b shows the 2D fluid velocity distribution in the different structures when the alkaline solution reached steady state. By contrast, the flow velocity inside the double-shell carbon model with stronger 2D mapping was obviously much faster than that of the single-shell carbon structure. The curves plotted by the line data from A to B illustrated that the maximum value of the flow velocity was calculated to be 0.8 × 10^–2^ m/s inside the double-shell carbon model, which was 20 times higher than 0.04 × 10^–2^ m/s in the single-shell carbon structure (Figure 5c,d). The vortex distributions in the different models were further investigated to examine the structure effect. As shown in Figure 5e,f, the double-shell carbon model presented a much stronger vortex inside the shells in comparison with the single-shell carbon structure, illustrating that the double-shell configuration could be conducive to the quick contact between electrolyte and electrode, thereby promoting the mass diffusion at the mesoscale.

**Figure 5:**
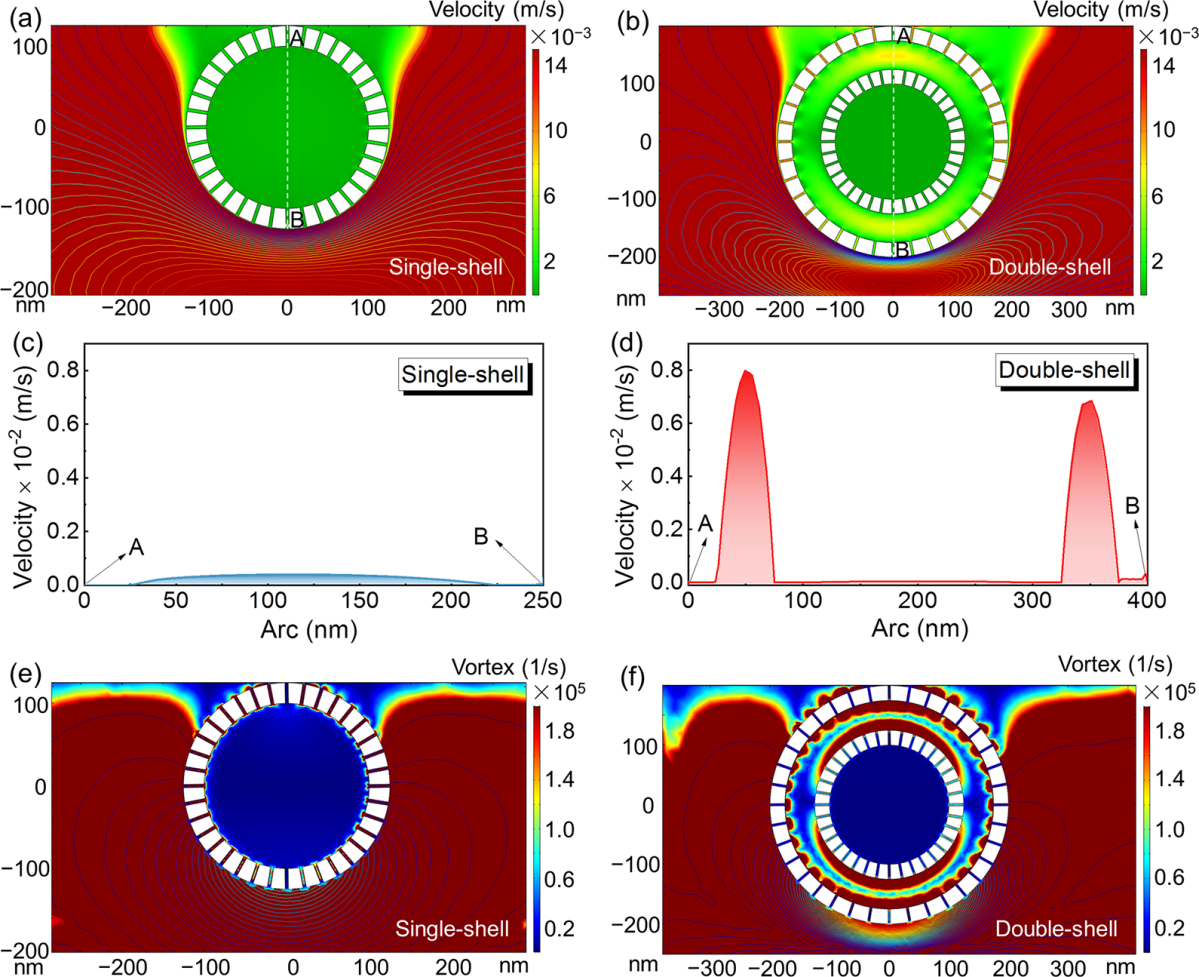
FEA simulation results. (a, b) The velocity fields and streamline distributions and the corresponding (c, d) velocity plots from A to B in the single-shell and double-shell models. (e, f) The 2D mapping images of the vortex fields in the single-shell and double-shell models.

In summary, we have precisely designed a group of hollow Ru-based nanoreactors by developing a double-shell hollow interlayer confinement strategy for boosting the HER process. Interestingly, the spatial position of Ru NPs can be finely modulated onto the single- and double-shell carbon spheres via altering the Ru^3+^ adsorption process. The middle shell not only completely separates the two carbon shells but also guarantees the formation and exposure of ultrafine Ru NPs due to the confinement effect. Moreover, the Ru species in the double-shell confinement environment avoids the thermal aggregation of Ru during pyrolysis and the loss of Ru active sites during the HER process. DFT calculations verify that the electronic structures are efficiently optimized, endowing Ru-DSC-I with improved conduction, H* desorption, and charge transfer, which lowers the HER energy barriers. FEA results indicate that the mesoscale diffusion of the electrolyte is highly promoted in the double-shell structure. Benefiting from the special spatial position of Ru in the hollow nanoreactor, Ru-DSC-I exhibits much better HER activity and stability than Ru-DSC-E and Ru-SSC. Our work provides a double-shell confinement design protocol for investigating the correlation of structure and properties toward electrocatalysis.

## Supporting Information Available

Experimental procedures, fabrication and electrochemical measurement of the electrodes, including Tafel slopes, ECSA curves, and EIS curves; characterization, including TEM images, SEM images, HRTEM images, XRD patterns, and XPS spectra; and DFT calculations, FEA simulations, and the additional tables (PDF)
