# Supplementary material for: Enabling Ultrafine Ru Nanoparticles with Tunable Electronic Structures via a Double-Shell Hollow Interlayer Confinement Strategy toward Enhanced Hydrogen Evolution Reaction Performance
Source: Nano Lett. 2023 Dec 1;24(2):592–600. doi: 10.1021/acs.nanolett.3c03514 (PMC10797610; doi:10.1021/acs.nanolett.3c03514)
Supplement: Supplementary file 1 — nl3c03514_si_001.pdf [file nl3c03514_si_001.pdf]

Supporting Information

Enabling Ultrafine Ru Nanoparticles with Tunable Electronic Structures via a Double-Shell Hollow Interlayer Confinement Strategy toward Enhanced Hydrogen Evolution Reaction Performance

Xiaoyan Liu[†,‡,§,※], Lihua Gong[†,※], Liwei Wang[‡], Chaoqun Chang[†], Panpan Su[‡], Yuhai Dou[#], Shi Xue Dou[#], Ying Li [§,*], Feilong Gong[†,*], and Jian Liu[‡,||,⊥,*]

[†] Key Laboratory of Surface and Interface Science and Technology of Henan Province, College of Material and Chemical Engineering, Zhengzhou University of Light Industry, Zhengzhou, Henan 450001, PR China

[‡] State Key Laboratory of Catalysis, Dalian Institute of Chemical Physics, Chinese Academy of Sciences, Dalian, Liaoning 116023, PR China

[§] Institute of Industrial Catalysis, Zhejiang University of Technology, Hangzhou, Zhejiang 310014, PR China

[||] DICP-Surrey Joint Centre for Future Materials, Department of Chemical and Process Engineering and Advanced Technology Institute of University of Surrey, Guildford, Surrey GU2 7XH, U.K.

[⊥] College of Chemistry and Chemical Engineering, Inner Mongolia University, Hohhot, Inner Mongolia 010021, PR China

[#] Institute of Energy Materials Science, University of Shanghai for Science and Technology, Shanghai 200093, PR China

[※] These authors are contributed equally to this work

*Corresponding author:

Jian Liu (jian.liu@surrey.ac.uk; jian.liu@imu.edu.cn); Feilong Gong (gfl@zzuli.edu.cn); Ying Li (liying@zjut.edu.cn)

Section S1. Experimental Procedures

Chemical and reagents

Chemicals: Styrene (AR), poly-vinypyrrolidone (PVP, K30), potassium persulfate (AR), hexadecyl trimethyl ammonium bromide (CTAB, 99%), 3-aminophenol (98%), formaldehyde aqueous solution (37% v/v), ammonia (NH_4OH, 28% in water), tetraethoxysilane (TEOS, AR), ruthenium (III) chloride ($RuCl_3 \cdot xH_2O$), Pt/C (20 wt.%, Alfa Aesar) and potassium hydroxide (KOH, AR) were used as received without further purification. Water was supplied from a Milli-Q water system (18.3 MΩ cm).

1.1 Synthesis of polystyrene (PS) spheres

PS spheres were synthesized by emulsifier-free polymerization from the reference. In a typical synthesis, PVP (1.10 g) was dissolved in 100 mL water into a 250 mL three-neck flask with magnetic continuous stirring. Next, 13 mL styrene (the polymerization inhibitor was removed by neutral alumina) was added into the system for another 10 min stirring. Followed, potassium perdulfate aqueous solution (0.33g dissolved in 20 mL water) was added. Then, the flask kept stirring in 70 °C oil bath for 24 h. Finally, the PS sphere were prepared and stored in aqueous solution. The particle mean size is 200 nm.

1.2 Synthesis of PS@APF

In a typical synthesis, CTAB (0.15 g) was dissolved in 30 mL mixed water-ethanol solution (volume ratio=5:3). Then, ammonia aqueous solution was added and stirred at room temperature for 0.5 h, followed by addition of 1.3 mL PS solution (which contains 0.1 g PS solid spheres). After stirring for another 0.5 h, 3-aminophenol (0.04 g) was added into the suspension and stirring was continued for 0.5 h. Next, a solution of formaldehyde (0.05 mL) was added. The mixture was stirred for 3 h at room temperature and subsequently heated for 12 h at 100 °C under static conditions in a Teflon-autoclave. The solid product was recovered by centrifugation and dried at 50 °C under vacuum oven for 24 h.

1.3 Synthesis of PS@APF-Ru

In a typical synthesis, 0.1 g of PS@APF powder was dispersed in 5 mL of water and the mixture was kept stirring for 30 min. To this, a certain amount of $RuCl_3$ aqueous solution was added and the result mixture was stirred for 12 h at room temperature to make sure the Ru^{3+} combine with $-NH_2$ group. This is followed by evaporation of water at an elevated temperature (50 °C) using rotary evaporator. The powder obtained was denoted as PS@APF-Ru.

1.4 Synthesis of Ru-DSC-I and Ru-DSC-E

In a typical synthesis of Ru-DSC-I, CTAB (0.10 g) was dissolved in a mixture of water (20 mL) and ethanol (8 mL). Then, 0.2 mL ammonia aqueous solution was added and stirred at room temperature for 0.5 h, followed by addition of 0.10 g of PS@APF-Ru. After stirring for 0.5 h, 200 uL TEOS was added, and the mixture was kept stirring for 12 h. Followed hydrothermal at 100 °C for 12 h. Next, the solid was recovered by centrifugation and washed with water and ethanol twice. And then, the products were dispersed in the solution again (which was mixed of 20 mL water, 8 mL ethanol, 0.2 mL NH_4OH and 0.10 g CTAB). After stirring for another 0.5 h, 3-aminophenol (0.05 g) was added into that suspension and stirring was continued for an additional 0.5 h. Next, a solution of formaldehyde (0.06 mL) was added. The mixture was stirred for 24 h at room temperature and subsequently heated for 12 h at 100 °C under static conditions in a Teflon-lined autoclave. The solid product was recovered by centrifugation and dried at 100 °C for 24 h. Finally, the powder was carbonization at 700 °C for 3 h with a rate at 1 °C min^{-1} under purity nitrogen (50 mL min^{-1}). And the PS core was decomposed completely during the carbonization process, however the SiO_2 shell need remove by NaOH. Finally, the obtained black powder was treated with 5% H_2/Ar atmosphere at 400 °C for 2 h to make the Ru ultrafine nanoparticles exposed. For the synthesis of Ru-DSC-E, the only difference is that PS@APF was used instead of PS@APF-Ru as the core, and $RuCl_3$ aqueous solution was added after the second APF shell coating.

1.5 Synthesis of Ru-SSC

In a typical synthesis, the pre-material preparation was finished after SiO_2 coating. And the PS@APF-Ru@SiO_2 was carbonized at 700 °C for 3 h with a rate at 1

°C min^{-1} under purity nitrogen (50 mL min^{-1}). After SiO_2 shell etching by NaOH solution, the obtained black powder was denoted as Ru-SSC.

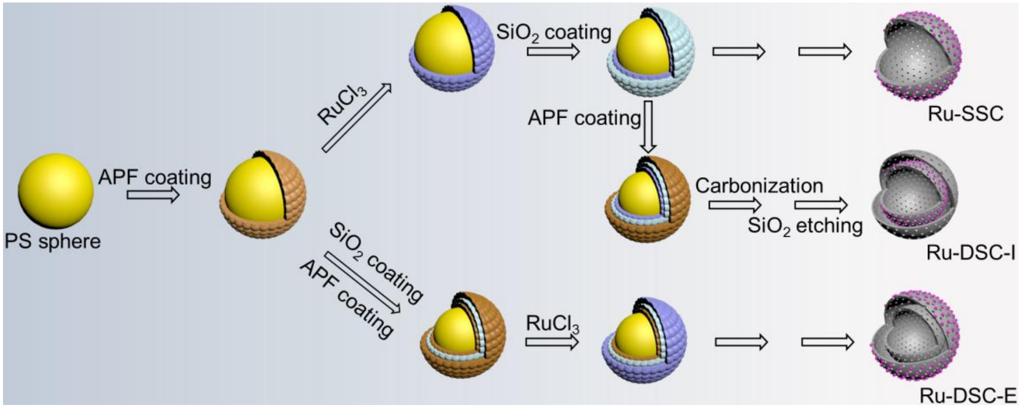

Scheme S1. Preparation diagram and morphology characterizations. (a) Schematic illustration of the preparation process of Ru-SSC, Ru-DSC-I, and Ru-DSC-E.

Section S2. Fabrication and Electrochemical measurement of the electrodes.

The electrochemical measurements were carried out at room temperature in a three-electrode glass cell connected to an electrochemical workstation (HCI 710e, CH, and Shanghai). To prepare working electrode, 2 mg of the as-prepared catalyst was dispersed in 830 μL water and 170 μL isopropanol with the assistance of ultrasonic for 0.5 h to form a homogeneous catalyst ink. Then 6 μL of the catalyst ink was dropcast onto a clean glassy carbon disk electrode (surface area 0.196 cm^2) at a loading of 0.17 mg cm^{-2}. After drying under an IR lamp, another 6 μL of 0.5 wt.% Nafion aqueous solution was cast onto the catalyst. After drying again, the catalytic working electrode can be used for the electrochemical study. For commercial Pt/C catalyst (Pt content: 20 wt.%), 1.8 mg sample was added to 3320 μL water and 680 μL isopropanol mixture to prepare the catalyst ink, and 2 μL of the catalyst ink was used for electrode preparation at a loading of 0.0127 mg cm^{-2}. Therefore, the metal loading of Ru and Pt in the electrode is at the same level, that is, Ru ($0.17 \times 1.5\% = 0.00255$ mg cm^{-2}) = Pt ($0.0127 \times 20\% = 0.00254$ mg cm^{-2}). A graphite rod and a Hg/HgO (1 M KOH) electrode were used as counter and reference electrode, respectively. Freshly prepared 1 M KOH aqueous solution (60 mL) was used as the electrolyte. The stability of the electrode was measured by testing the CV for 500 cycles. IR-compensation was set at 95% of solution resistance in all measurements. The potential was calibrated versus

reversible hydrogen electrode (RHE) using the following equation:

$$E\ (RHE) = E\ (Hg/HgO) + 0.059*pH + 0.098$$

The Tafel slope was calculated by fitting the linear portion of the Tafel plots following the Tafel equation:

$$\eta = b\ \log(j)+a$$

The electrochemical active surface area (ECSA) calculation of the catalysts was based on the following equation:

$$ECSA = Cdl/Cs$$

Where the Cs represent the specific capacitance that is 0.04 mF cm^{-2} in alkaline solution.

Section S3. Characterization

High resolution transmission electron microscopy (HRTEM) measurements were conducted on a JEM-200F microscope (JEOL, Tokyo, Japan) operated at 200 kV. The samples for TEM measurement were suspended in ethanol and supported onto a holey carbon film on a Cu grid. The nitrogen-sorption experiments were performed at −196 °C on a Micromeritics Tristar 3020 (Atlanta, GA, USA) system with mesoporous analysis. Before the measurement, the samples were out-gassed at 300 °C for 8 h. The Brunauer-Emmett-Teller specific surface areas were calculated using adsorption data at a relative pressure range of P/P_0=0.05-0.25. Pore size distribution was investigated by Barreet-Joyner-Halenda (BJH) method. X-ray diffraction (XRD) patterns were acquired Cu-Kα (λ=1.5418) in the scan range of 10 to 90 °C at a step of 5 °C min^{-1}. Raman spectra were collected on a xx Raman spectrometer with 532 nm excitation laser at a power of 0.7 mW. The contact angles (CAs) of water of the Ru-DSC-I and Ru-DSS were tested at room temperature. The thermal property of each sample in N_2 was determined by thermogravity-differential scanning calorimetry (TG-DSC) on a xx instrument. The chemical composition of the catalyst was analyzed by inductively coupled plasma atomic emission spectroscopy (ICP-AES) using an xx spectrometer. CO chemisorption was used to determine the dispersion of Ru by using a xx instrument. The weighed sample (~100 mg) was purged with He for 30 min at room temperature and reduced at 200 °C for 2 h under. X-ray photoelectron spectroscopy

(XPS) were collected on a Thermo Scientific Escalab (ESCALAB 250Xi, Massachusetts) with monochromatized Al Kα X-ray as the excitation source.

Section S4. DFT calculation

The Vienna Ab initio Simulation Package (VASP) was employed to study all the density functional theory (DFT) calculations. The projector augmented wave method with Perdew-Burke-Ernzerh of pseudopotentials was utilized to treat the electron-ion interaction. The plan wave basis was set as 400 eV. The total energy convergence criterion of electronic self-consistent field (SCF) was carried out 10^{-5} eV while the structural optimization was relaxed until the force was less than 0.05 eV/Å. Then the spin polarization was adopted. The Brillouin zone in the calculation was sampled by a 3×3×1 k points mesh. The vacuum space along the Z direction was 15 Å to prevent the effect of the adjacent atomic slabs. As shown in Figure S15, the models that Ru were onto the surface of carbon (Ru@C-S) and between the double-layer carbon (Ru@C-I) were built, respectively.

Section S5. FEA simulation

The "Laminar Flow" module of COMSOL Multiphysics solver was used to simulate the structure effect of the materials, and the corresponding 2D mode was collected. According to the experimental data, the single-shell carbon model was built with a diameter of 200 nm, a shell thickness of 25 nm, and a pore diameter of *ca.* 3 nm. The double-shell carbon model (Figure 7b) was plotted with two circles which a diameter of 200 nm and 350 nm, each shell thickness was 25 nm and the distance of the two shells was 50 nm, the pore size was *ca.* 3 nm. The calculated domain was simulated via filling the electrolyte 1 M KOH in a rectangular region (6000w nm × 6010h nm). The electrolyte was poured from the left boundary, and outflowed from right boundary. The inflow velocity was set to 0.25 m s^{-1} at the entrance. The flow field was done on the "Laminar Flow" module. In order to represent the adhesion of the materials on electrode, the calculated models were put on the coboundary of the domain in the stationary simulation. The flow field was calculated by the Navier-Stokes equations:

$$\nabla \cdot \mathrm{u} = 0 \qquad (1)$$

$$\rho(u \cdot \nabla)u = \nabla \cdot [-pI + \mu(\nabla u + (\nabla u)^T)] + F \quad (2)$$

Where the u, ρ, p, **μ** and F represent the flow velocity, fluid density, pressure, dynamic viscosity and volume force vector, respectively.

Section S6. Supporting Figures

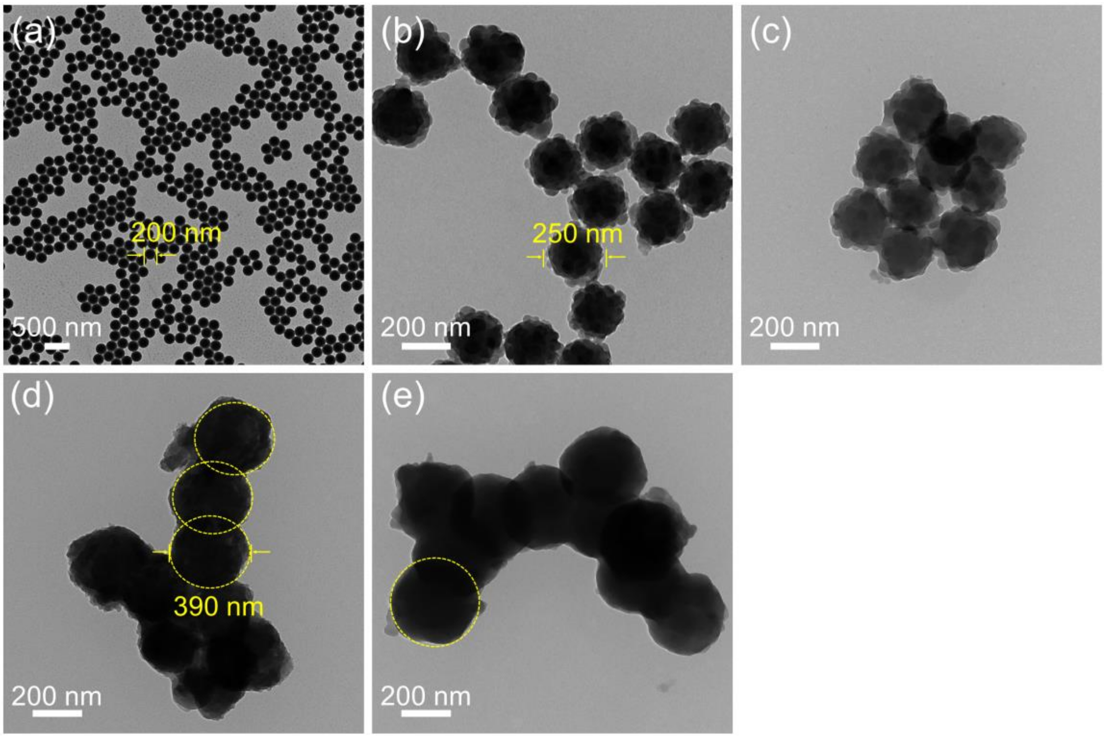

Figure S1. (a) TEM images of the PS spheres, (b) PS@APF spheres, (c) PS@APF-Ru composites, (d) PS@APF@SiO_2 composites, and (e) PS@APF@SiO_2@APF composites.

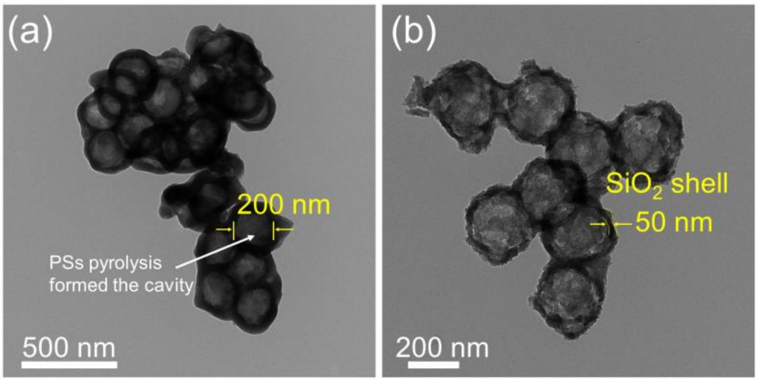

Figure S2. Calcination product of the PS@APF@SiO_2@APF composites in (a) N_2 and (b) Air, respectively.

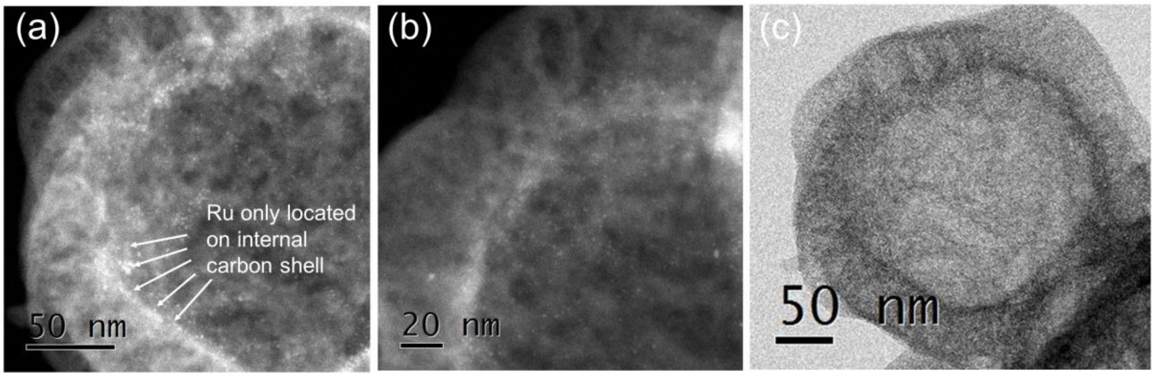

Figure S3. (a-b) HAADF-HRTEM and enlarged images, (c) HRTEM image of the Ru-DSC-I.

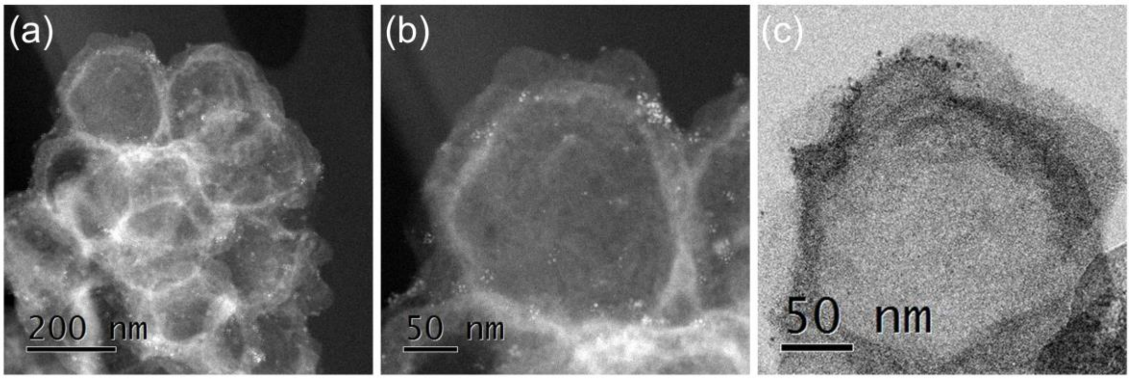

Figure S4. (a-b) HAADF-HRTEM and enlarged images, (c) HRTEM image of the Ru-DSC-E.

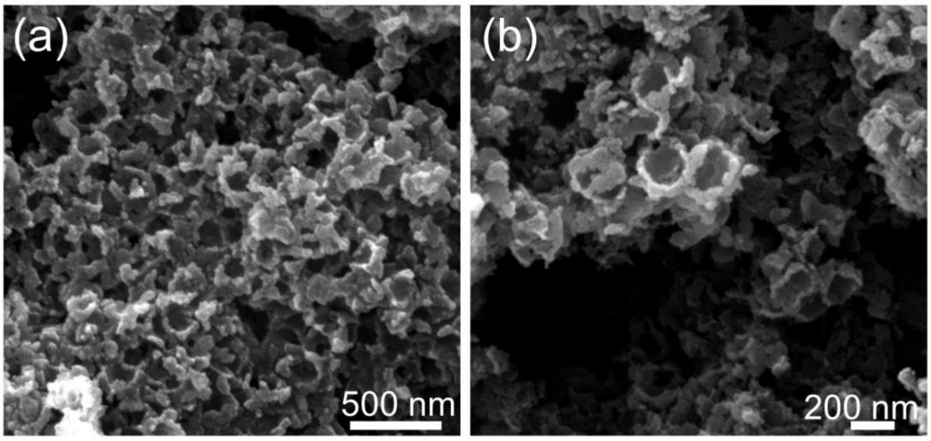

Figure S5. SEM and enlarged SEM images of the Ru-SSC.

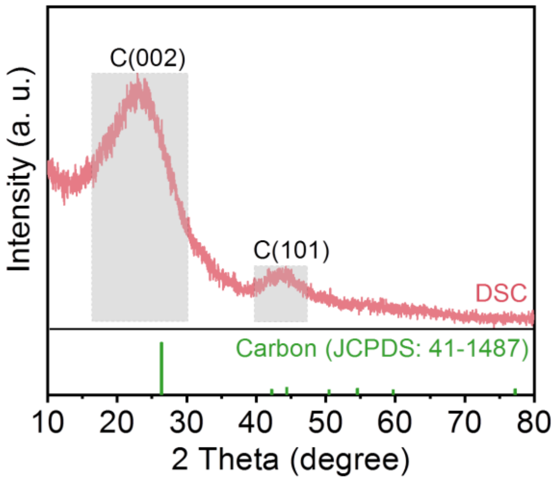

Figure S6. XRD pattern for the DSC.

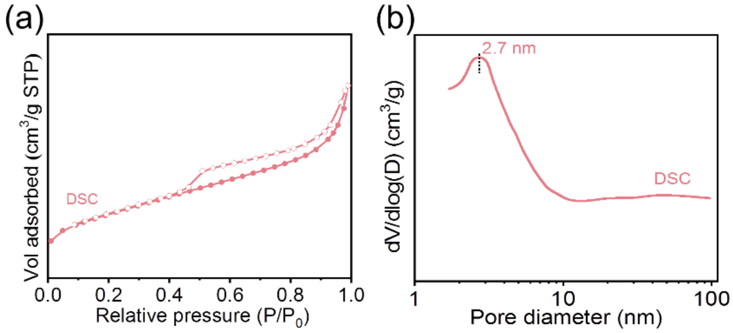

Figure S7. (a) N_2 adsorption-desorption isotherms and (b) pore diameter distribution of the DSC.

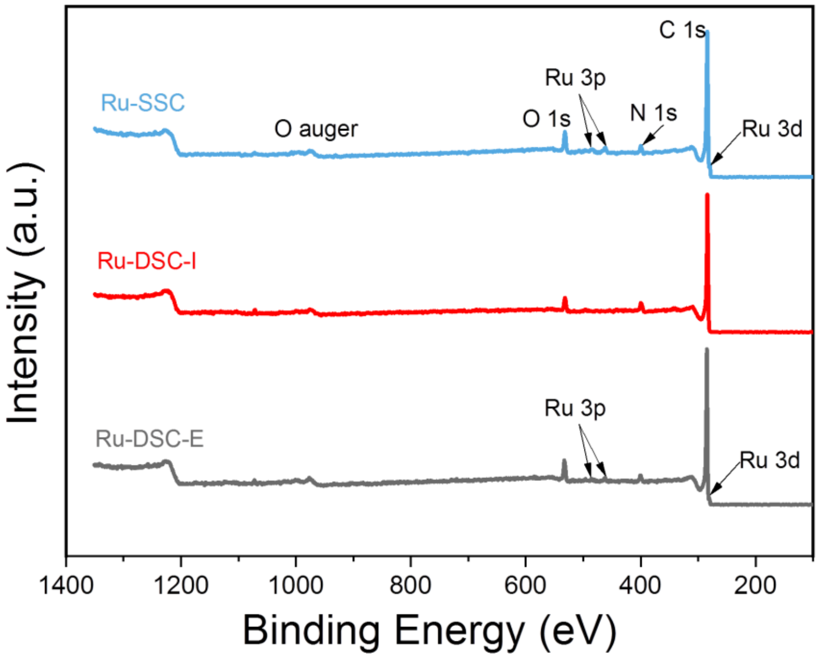

Figure S8. The comparison of XPS spectra of survey for the Ru-SSC, Ru-DSC-I, and Ru-DSC-E.

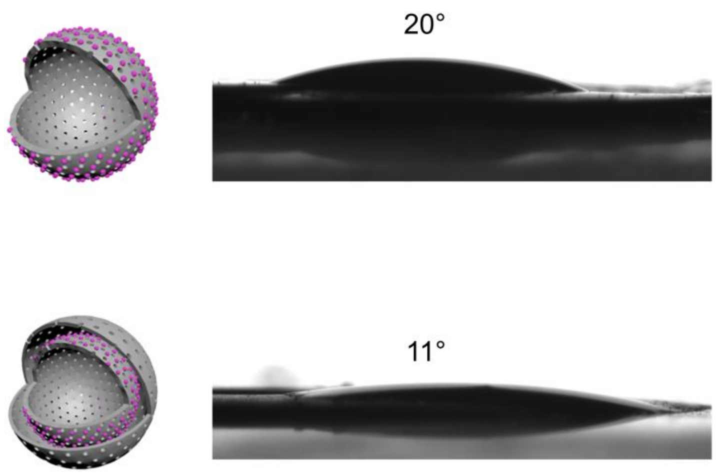

Figure S9. Contact angle of the water droplets for Ru-SSC and Ru-DSC-I.

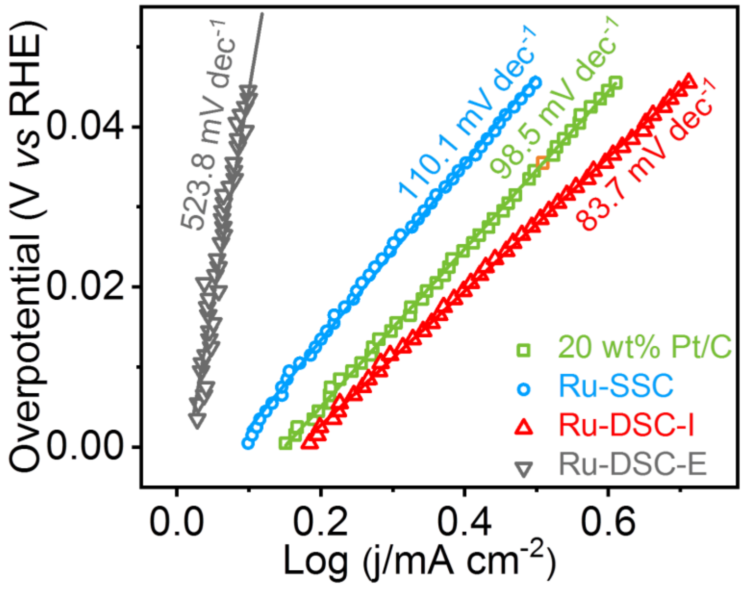

Figure S10. The Comparison of Tafel slopes for the 20 wt% Pt/C, Ru-SSC, Ru-DSC-I, and Ru-DSC-E.

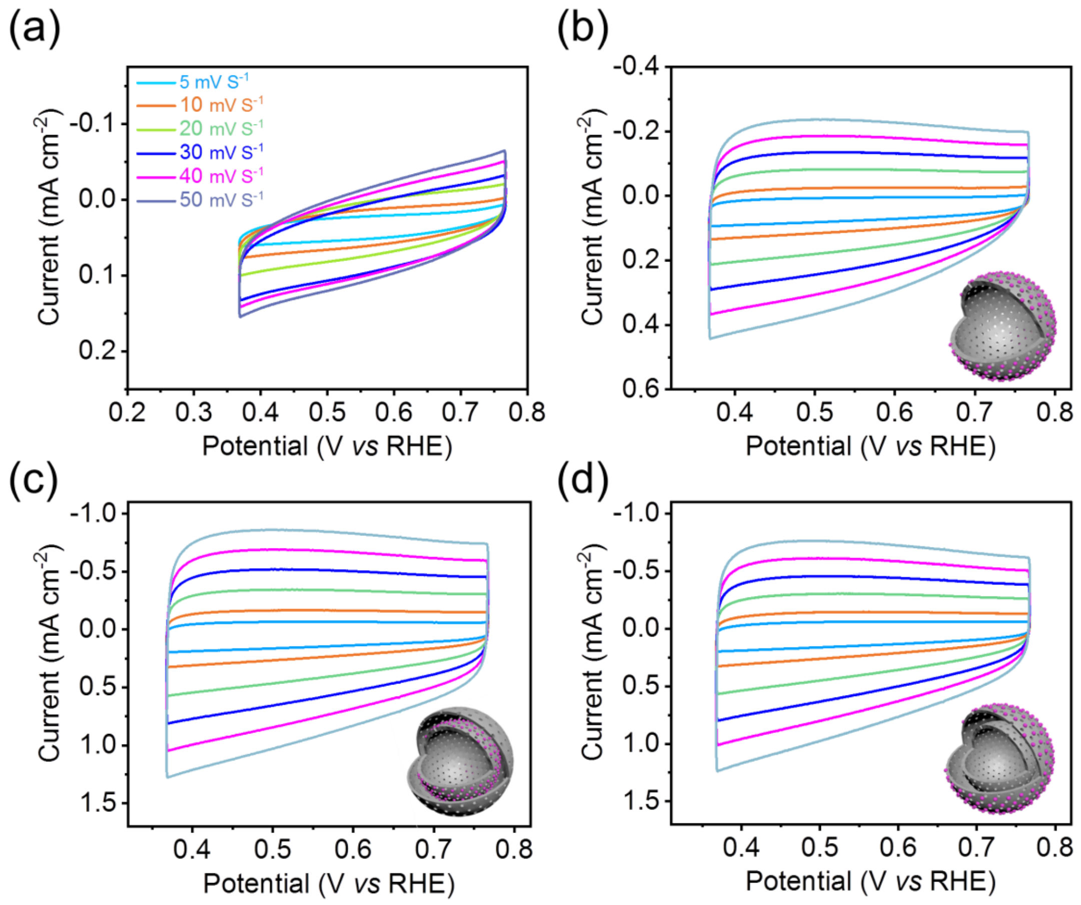

Figure S11. ECSA curves of the (a) Ru-SSC, (b) Ru-DSC-I and (c) Ru-DSC-E with a series scan rate at 5, 10, 20, 30, 40, and 50 mV s^{-1}.

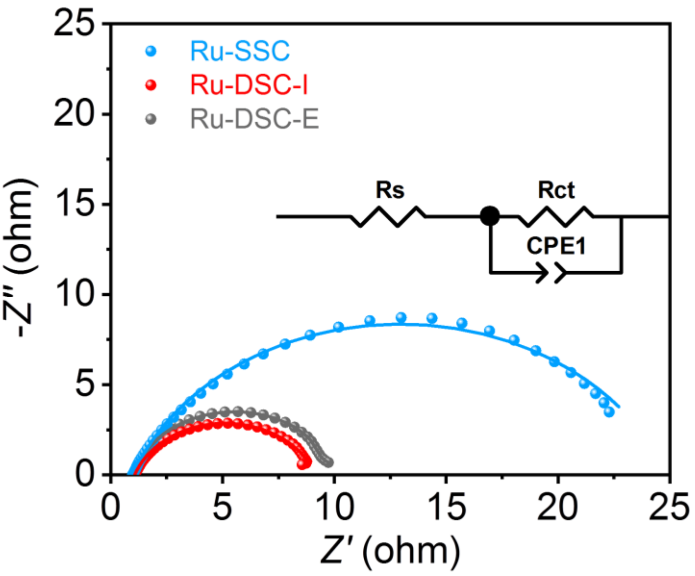

Figure S12. The EIS curves and corresponding equivalent circuit model for the Ru-SSC, Ru-DSC-I, and Ru-DSC-E at open-circuit voltages.

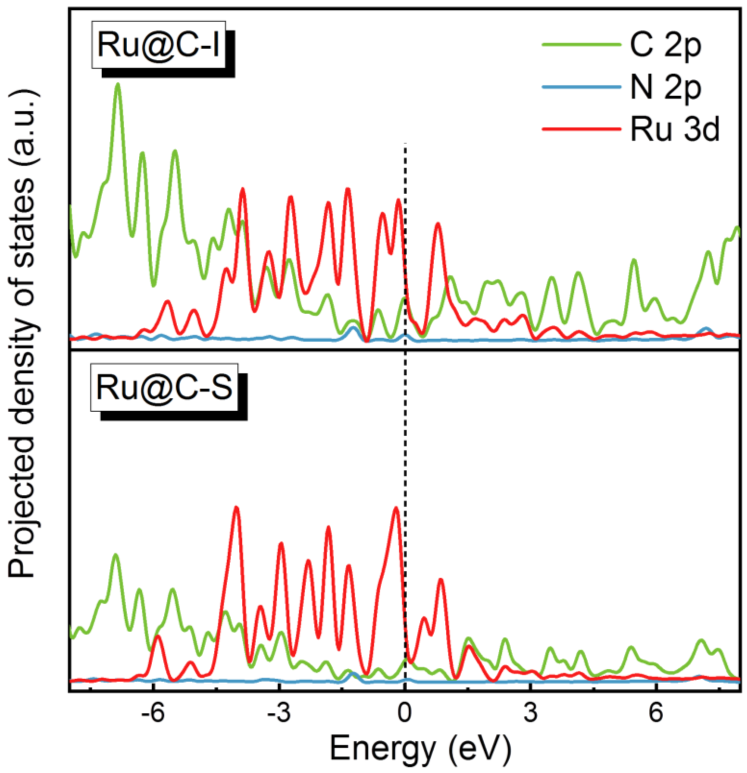

Figure S13. The comparison of PDOS for the Ru@C-S and Ru@C-I.

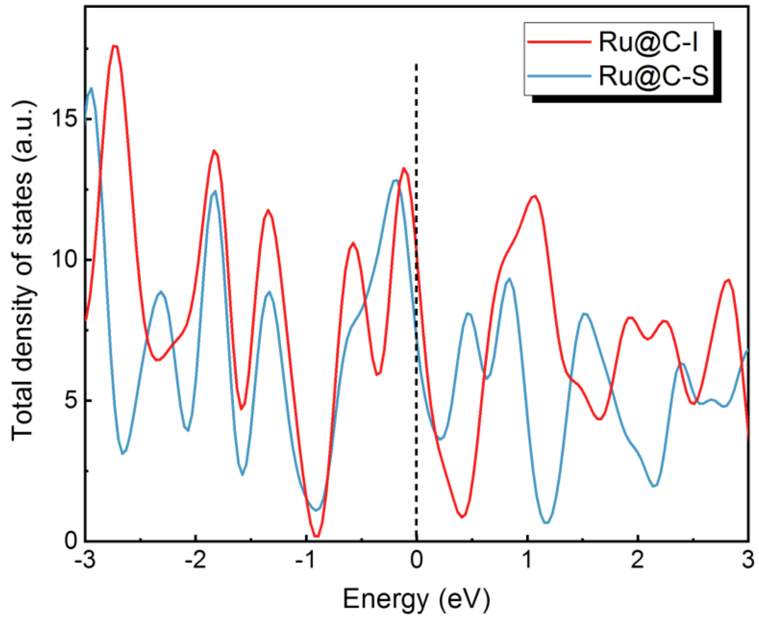

Figure S14. The comparison of TDOS for the Ru@C-S and Ru@C-I.

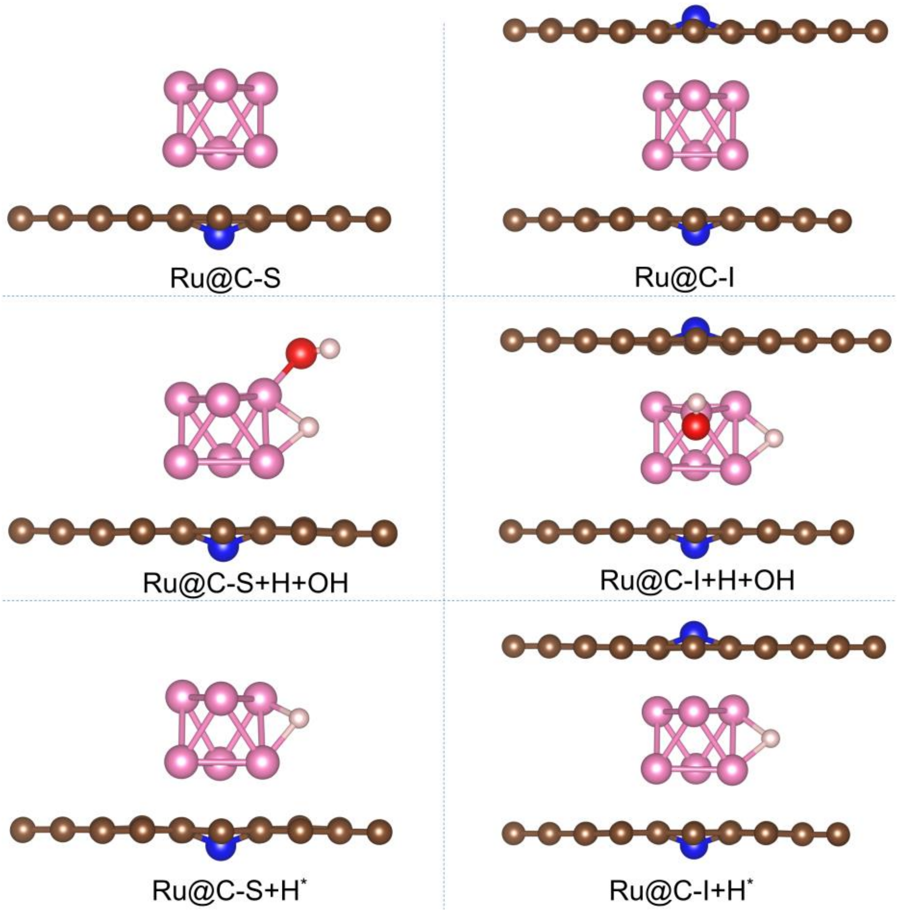

Figure S15. The optimized structures of the Ru@C-S and Ru@C-I before and after adsorbing intermediates.

Section S7. Supporting Tables

Table S1. Texture properties of as-prepared catalysts.

Entry	Catalyst	Ru (wt%)[a]	S. A. (m^2 g^{-1})[b]	P. V. (cm^3 g^{-1})[c]	Pore size (nm)[d]	Elemental analysis			
						C (wt.%)	N (wt.%)	O (wt.%)	H (wt.%)
1	DSC	-	779	1.04	2.7	-	-	-	-
2	Ru-SSC	1.37	494	0.71	2.1	84.0	2.5	11.9	1.6
3	Ru-DSC-I	1.50	631	0.55	2.4	84.0	5.3	9.4	1.4
4	Ru-DSC-E	1.58	676	0.66	2.4	84.4	4.4	9.7	1.5

[a] Ru content is detected by ICP-AES.

[b] BET surface.

[c] Total volume.

[d] Pore diameter calculated from adsorption branch of the isotherm.

Table S2. The comparison of HER overpotentials for the Ru-DSC-I and catalysts reported in the literature. (Current density: 10 mA cm^{-2}; electrolyte: 1 M KOH)

Catalysts	η_{10}	Ref.
Ru/$Y(OH)_3$ NHs 0.1 M KOH	100	1
$Cu_{2-x}S$@Ru NPs	82	2
Ru-MoO_2-800 °C	74	3
RuO_2/Co_3O_4	89	4
1D-RuO_2-CNx 0.5M KOH	95	5
Rh-CoNi LDH/MXene	74.6	6
Ru-$Ti_3C_2T_x$@600	96	7
RuNC-2	81	8
$SrRuO_3$	110	9
Ru/Mo_2CT_x	78	10
Ru-WSe_2	87	11
SA-Ru-MoS_2	76	12
Ru/Ni_2P NPs	132	13
Ru BCNNs/PNC NNSs	81.3	14
Ru/C_3N_4/C 0.1 M KOH	79	15
**Ru-DSC-I**	**73.5**	**This work**

Table S3. ICP-AES of as-prepared Ru-based electrocatalysts after catalysis.

Entry	catalyst	Ru (wt.%)[a]
1	Ru-SSC	0.83
2	Ru-DSC-I	1.47
3	Ru-DSC-E	0.96

[a] Ru content is detected by ICP-AES.

References

(1) Liu, Y.; Lu, X.; Che, Z.; Zhang, C.; Han, M.; Bao, J.; Dai, Z. Amorphous $Y(OH)_3$-promoted $Ru/Y(OH)_3$ nanohybrids with high durability for electrocatalytic hydrogen evolution in alkaline media. *Chem. Commun.* **2018**, *54* (86), 12202-12205.

(2) Yoon, D.; Lee, J.; Seo, B.; Kim, B.; Baik, H.; Joo, S. H.; Lee, K. Cactus-Like Hollow Cu2-xS@Ru Nanoplates as Excellent and Robust Electrocatalysts for the Alkaline Hydrogen Evolution Reaction. *Small* **2017**, *13* (29), 1700052.

(3) Koo, Y.; Oh, S.; Im, K.; Kim, J. Ultrasonic spray pyrolysis synthesis of nano-cluster *APF-derived* on molybdenum dioxide for hydrogen evolution reaction. *Appl. Surf. Sci.* **2023**, *611*, 155774.

(4) Liu, H.; Xia, G.; Zhang, R.; Jiang, P.; Chen, J.; Chen, Q. MOF-derived RuO2/Co3O4heterojunctions as highly efficient bifunctional electrocatalysts for HER and OER in alkaline solutions. *RSC Adv.* **2017**, *7* (7), 3686-3694.

(5) Bhowmik, T.; Kundu, M. K.; Barman, S., Growth of One-Dimensional RuO_2 Nanowires on g-Carbon Nitride: An Active and Stable Bifunctional Electrocatalyst for Hydrogen and Oxygen Evolution Reactions at All pH Values. *ACS Appl. Mater. Inter.* **2016**, *8* (42), 28678-28688.

(6) Yan, L.; Song, D.; Liang, J.; Li, X.; Li, H.; Liu, Q. Fabrication of highly efficient Rh-doped cobalt–nickel-layered double hydroxide/MXene-based electrocatalyst with rich oxygen vacancies for hydrogen evolution. *J. Colloid Interf. Sci.* **2023**, *640*, 338-347.

(7) Liu, T.; Zhang, W.; Chen, T.; Liu, D.; Cao, L.; Ding, T.; Liu, X.; Pang, B.; Wang, S.; Wang, L.; Luo, Q.; Yao, T. Regulating the Coordination Environment of Ruthenium Cluster Catalysts for the Alkaline Hydrogen Evolution Reaction. *J. Phys. Chem. Lett.* **2021**, *12* (33), 8016-8023.

(8) Li, Y.; Chu, F.; Liu, Y.; Kong, Y.; Tao, Y.; Li, Y.; Qin, Y. An ultrafine ruthenium nanocrystal with extremely high activity for the hydrogen evolution reaction in both acidic and alkaline media. *Chem. Commun.* **2018**, *54* (93), 13076-13079.

(9) Sugawara, Y.; Kamata, K.; Yamaguchi, T. Extremely Active Hydrogen Evolution Catalyst Electrochemically Generated from a Ruthenium-Based Perovskite-Type Precursor. *ACS Appl. Energ. Mater.* **2019**, *2* (2), 956-960.

(10) Wu, Y.; Wang, L.; Bo, T.; Chai, Z.; Gibson, J. K.; Shi, W. Boosting Hydrogen Evolution in Neutral Medium by Accelerating Water Dissociation with Ru Clusters Loaded on Mo_2CTx MXene. *Adv. Funct. Mater.* **2023**, *33* (16).

(11) Zhao, Y.; Mao, G.; Huang, C.; Cai, P.; Cheng, G.; Luo, W. Decorating WSe_2 nanosheets with ultrafine Ru nanoparticles for boosting electrocatalytic hydrogen evolution in alkaline electrolytes. *Inorg. Chem. Front.* **2019**, *6* (6), 1382-1387.

(12) Zhang, J.; Xu, X.; Yang, L.; Cheng, D.; Cao, D. Single-Atom Ru Doping Induced Phase Transition of MoS_2 and S Vacancy for Hydrogen Evolution Reaction. *Small Methods* **2019**, *3* (12), 1900653.

(13) Chi, J.-Q.; Zhang, X.-Y.; Ma, X.; Dong, B.; Zhang, J.-Q.; Guo, B.-Y.; Yang, M.; Wang, L.; Chai,

Y.-M.; Liu, C. Interface Charge Engineering of Ultrafine Ru/Ni2P Nanoparticles Encapsulated in N,P-Codoped Hollow Carbon Nanospheres for Efficient Hydrogen Evolution. *ACS Sustain. Chem. Eng.* **2019**, *7* (21), 17714-17722.

(14) Xu, G.-R.; Jiang, X.; Sun, T.; Wang, X.; Li, B.; Wu, Z.; Liu, H.; Wang, L. Ru branched nanostructure on porous carbon nanosheet for superior hydrogen evolution over a wide pH range. *J. Alloys* Compd. **2023**, *947*, 169393.

(15) Zheng, Y.; Jiao, Y.; Zhu, Y.; Li, L. H.; Han, Y.; Chen, Y.; Jaroniec, M.; Qiao, S.-Z. High Electrocatalytic Hydrogen Evolution Activity of an Anomalous Ruthenium Catalyst. *J. Am. Chem. Soc.* **2016**, *138* (49), 16174-16181.
